# Virulence Profiles of *Vibrio vulnificus* in German Coastal Waters, a Comparison of North Sea and Baltic Sea Isolates

**DOI:** 10.3390/ijerph121215031

**Published:** 2015-12-15

**Authors:** Nadja Bier, Claudia Jäckel, Ralf Dieckmann, Nicole Brennholt, Simone I. Böer, Eckhard Strauch

**Affiliations:** 1 National Reference Laboratory for Monitoring Bacteriological Contamination of Bivalve Mollusks, Department of Biological Safety, Federal Institute for Risk Assessment, Max-Dohrn-Str. 8-10, Berlin D-10589, Germany; nadja.bier@bfr.bund.de (N.B.); claudia.jaeckel@bfr.bund.de (C.J.); ralf.dieckmann@bfr.bund.de (R.D.); 2 Federal Institute of Hydrology, Am Mainzer Tor 1, Koblenz D-56068, Germany; Brennholt@bafg.de (N.B.); simone_boeer@hotmail.com (S.I.B.)

**Keywords:** multilocus sequence typing, virulence-associated traits, genotypes, pathogenicity potential, vibrio infection, public health risk, global warming

## Abstract

*Vibrio vulnificus* is a halophilic bacterium of coastal environments known for sporadically causing severe foodborne or wound infections. Global warming is expected to lead to a rising occurrence of *V. vulnificus* and an increasing incidence of human infections in Northern Europe. So far, infections in Germany were exclusively documented for the Baltic Sea coast, while no cases from the North Sea region have been reported. Regional variations in the prevalence of infections may be influenced by differences in the pathogenicity of *V. vulnificus* populations in both areas. This study aimed to compare the distribution of virulence-associated traits and genotypes among 101 *V. vulnificus* isolates from the Baltic Sea and North Sea in order to assess their pathogenicity potential. Furthermore, genetic relationships were examined by multilocus sequence typing (MLST). A high diversity of MLST sequences (74 sequence types) and differences regarding the presence of six potential pathogenicity markers were observed in the *V. vulnificus* populations of both areas. Strains with genotypes and markers associated with pathogenicity are not restricted to a particular geographic region. This indicates that lack of reported cases in the North Sea region is not caused by the absence of potentially pathogenic strains.

## 1. Introduction

### 1.1. Background

*Vibrio vulnificus* belongs to the family of *Vibrionaceae* and is ubiquitously found in coastal, estuarine, and brackish environments in the water column, in sediments, as well as in or associated to fish, bivalve mollusks, crustaceans, and plankton [1,2]. The species causes foodborne infections resulting either in mild gastroenteritis with diarrhea, vomiting and abdominal pain or life-threatening primary septicemia, with mortality rates of 61% [3]. *V. vulnificus* can also enter the body through preexisting wounds exposed to seawater or through skin lesions incurred by handling of seafood or by fishing accidents. Due to the high multiplication rate of the pathogen, wound infections may quickly progress to necrotizing fasciitis and even secondary septicemia with mortality rates of 17% [3]. Regular alcohol abuse and liver diseases such as hemochromatosis, hepatitis, and cirrhosis are among the major risk factors for developing severe *V. vulnificus* infections, probably due to elevated serum iron levels in these patients [3,4].

In the United States, 43% of *V. vulnificus* infections manifest as primary septicemia and 45% as wound infections [3], whereby the incidence of wound infections is rising [5]. In contrast, foodborne infections are of minor significance in Germany, as reported cases occurred exclusively after contact to seawater [6]. Despite the high number of individuals with predisposing diseases (e.g., 36 million persons in the U.S.) and the great abundance of *V. vulnificus* in seawater and seafood, infections are only sporadically observed (about 100 reported cases annually in the U.S.) [7]. This indicates that so far unknown host factors may be essential in allowing infections in only a few people, and/or that only a small proportion of the *V. vulnificus* population is able to cause human infection [4]. Consequently, identification of putative virulence factors and pathogenicity markers are major targets of *V. vulnificus* research worldwide.

Several studies on multilocus sequence typing (MLST) of clinical and environmental *V. vulnificus* isolates showed a subdivision into two major lineages or clusters. Generally, most isolates within MLST cluster I were of environmental origin, while the majority of strains within cluster II were clinical isolates, thus implying a higher virulence potential for cluster II strains [8,9,10,11]. Molecular analyses revealed two alleles of the 16S rRNA gene (type A, type B) and of the so called “virulence correlated gene” of unknown function (*vcg*; type E and type C). 16S rRNA-type B and *vcg*-type C are correlated with increased mouse virulence and with the clinical origin of isolates [12,13,14,15]. Recent studies showed a high association of additional genes with clinical isolates from the Baltic Sea region and northeastern USA. These included the 33-kb genomic region XII, a mannitol fermentation operon, and the *nanA* gene encoding a *N*-acetylneuraminate lyase, the key enzyme for sialic acid catabolism [10,11,16]. This implies that these potential virulence markers should be additionally addressed when characterizing *V. vulnificus* pathogenicity.

*V. vulnificus* occurrence shows seasonal and regional variations with high temperature and low salinity as the major factors supporting growth [17,18]*.* The greater abundance of *V. vulnificus* in seawater and seafood in the summer months is reflected by increased incidences of human infection [19]. Especially in Northern Europe, occasional human infections have been linked to climate anomalies with most of the cases following heat waves, as documented for the years 2003, 2006 and 2010 [6,20,21,22,23]. Thus, impacts of climate change, such as rising sea surface temperatures and an increasing frequency of heat waves are assumed to favor proliferation and distribution of the pathogen in coastal areas at higher latitudes. This in turn may lead to higher incidences of *V. vulnificus* illness in Northern Europe [20,21,22,23]. Especially the Baltic Sea represents a high risk area, as it is a low salinity intercontinental sea and one of the fastest warming marine ecosystems on Earth [20]. So far, *V. vulnificus* infections in Germany have been documented for the Baltic Sea region, while no cases have been reported from the North Sea coast [6].

### 1.2. Aim of the Study

In view of climate projections and the demographic change, investigations on the pathogenicity potential of *V. vulnificus* isolates present in the North Sea and Baltic Sea are highly demanded as both regions represent popular tourist destinations. Variations in the prevalence of infections in both geographical regions may be influenced by a differing pathogenic potential of *V. vulnificus* populations. This study aimed to examine the genetic relationship of *V. vulnificus* isolates from both regions by MLST as well as the distribution of virulence-associated traits and genotypes to assess their pathogenic potential.

## 2. Experimental Section

### 2.1. Bacterial Strains

**Figure 1:**
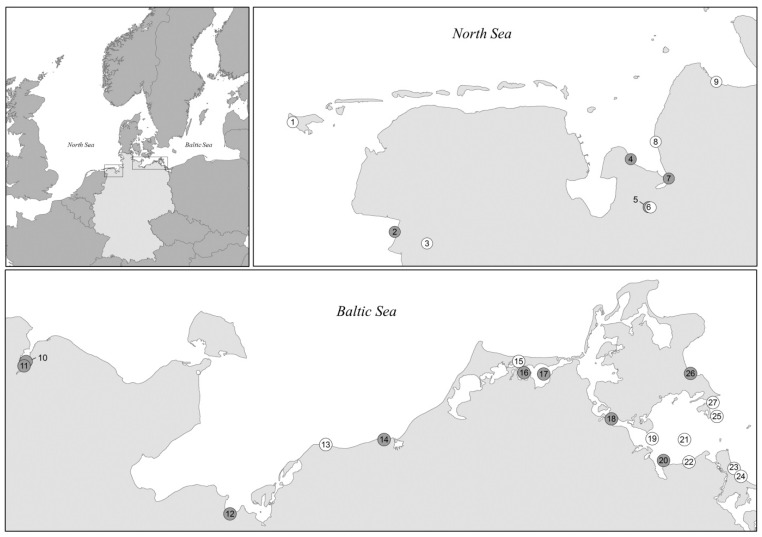
Geographical location of sampling sites along the North Sea and Baltic Sea coastline. For detailed information on sampling sites, refer to Supplementary Table S1. Grey dots indicate sampling sites at which strains of MLST cluster II have been isolated at least once. White dots represent sampling sites at which all isolated strains belonged to MLST cluster I. Sampling Sites 3, 5 and 6 are located in estuaries, rivers are not indicated in the map.

Multilocus sequence typing (MLST) and characterization of virulence-associated traits were conducted on a total of 101 *V. vulnificus* strains, including 50 strains originating from the North Sea and 51 strains recovered from the Baltic Sea. Strains were exclusively isolated from water (*n* = 56) and sediment samples (*n* = 45) collected between 2010 and 2012 (between May and October) by local health authorities. Sampling sites were located along the German Baltic Sea and North Sea coastline, as well as within the estuaries of the rivers Ems and Weser. Detailed information on the location and classification of the sampling sites is given in Figure 1 and Supplementary Table S1. The isolates derived from strain collections gathered within the German research programs KLIWAS and VibrioNet [6,18,23,24,25,26]. To achieve a diverse collection, isolates were chosen from each available sampling site and date. Strains were obtained from nine sampling sites in the North Sea on 13 different sampling dates and from 18 sampling sites in the Baltic Sea on 16 different sampling dates.

All isolates and detailed sampling information are listed in Supplementary Table S2. Species identities of all strains were confirmed by a species-specific *toxR* polymerase chain reaction (PCR) amplification as described previously [27]. In parallel, strains were identified by a matrix assisted laser desorption/ionization time of flight (MALDI-TOF) mass spectrometry analysis using the MALDI Biotyper System (Bruker Daltonic, Bremen, Germany) and the direct transfer method [28]. Bacterial strains were grown overnight on LB (Lysogeny broth) agar at 37 °C. Single colonies were transferred to a stainless steel target and overlaid with matrix solution (10 mg/mL α-cyano-4-hydroxycinnamic acid in acetonitrile, water, and trifluoroacetic acid, 50:47.5:2.5, *v*/*v*).

### 2.2. Biotyping and Mannitol Fermentation

Strains were characterized by a PCR described by Sanjuán *et al.* [29] allowing simultaneous amplification of target genes specific for *V. vulnificus*, biotype 2 and serovar E. Strains were further tested for the ability to cleave indole from tryptophan and to ferment sorbitol. *V. vulnificus* strains were streaked onto LB agar plates and incubated at 37 °C for 24 h. For indole reaction, 5 mL DEV-tryptophan-broth (0.1% tryptophan, 1% peptone, 0.5% NaCl, pH 7.2, Merck KGaA, Darmstadt, Germany) were inoculated with a single colony and incubated at 37 °C for 24 h. Generated indole was detected by dropping 100 µL Kovac’s reagent (Merck KGaA, Darmstadt, Germany) on top of the culture. A positive indole reaction resulted in the formation of a red colored upper phase. For sorbitol fermentation, 5 mL sorbitol fermentation broth (1% sorbitol, 0.0075% bromothymol blue, 1% peptone, 0.5% NaCl, pH 7.4) were inoculated, incubated at 37 °C, and examined for fermentation after 24 h and five days indicated by a color change from blue/green to yellow. *V. vulnificus* strains were tested for mannitol fermentation analogously to the sorbitol fermentation test with 1% mannitol, as described previously [11].

### 2.3. Multilocus Sequence Typing

MLST analysis was performed based on ten housekeeping genes (*glp*, *gyrB*, *mdh*, *metG*, *purM*, *dtdS*, *lysA*, *pntA*, *pyrC*, *tnaA*) as described by Bisharat *et al.* [8,9] using primers and protocols published on the *V. vulnificus* Multilocus Sequence Typing website [30]. Electropherograms were assembled and trimmed using SeqMan Pro (v12; DNASTAR Lasergene, Madison, WI, USA) and Accelrys Gene (v2.5, Accelrys Inc., San Diego, CA, USA). Allele sequences at each locus and corresponding allelic profiles were compared to the PubMLST database. New allele sequences and allelic profiles (sequence types, STs) were submitted to the PubMLST database. A list of all strains with corresponding allelic profiles is provided in Supplementary Table S3. To elucidate clonal relationships, allelic profiles were analyzed with the PHYLOViZ software using the goeBURST algorithm [31]. Related strains with allelic profiles differing in one of ten loci (single locus variants, SLV) were assigned to a distinct clonal complex. The PHYLOViZ software was used to generate a full Minimum Spanning Tree (MST) displaying the relationships between STs of the whole dataset. Allele sequences were concatenated in the order of loci used to define the allelic profile to generate a 4326 bp concatemer for each strain. A phylogenetic tree was constructed in MEGA ver. 6 [32] based on the alignment of concatenated allele sequences using the Unweighted Pair Group Method with Arithmetic Mean (UPGMA) [33]. Distances were computed using the Maximum Composite Likelihood method [34] and the reliability of the tree was assessed by bootstrap analysis with 1000 replicates. Sequences from clinical and environmental biotype 1 isolates from the Baltic Sea region from our previous study [11] were included in the analysis for comparison. The same procedure was performed on concatemers of 1299 bp obtained by concatenating the sequences of three housekeeping genes in the order *gyrB*, *dtdS* and *pyrC*.

### 2.4. PCR Analyses

The RTP Bacteria DNA Kit (STRATEC Biomedical AG, Birkenfeld, Germany) was used for extraction of genomic DNA. PCR amplifications were carried out on a Mastercycler EP Gradient (Eppendorf, Hamburg, Germany) in a volume of 25 µL with 1× PCR-buffer (2 mM MgCl_2_), 0.2 mM of each dNTP, 0.2 µM of each primer, and 1.5 U DreamTaq DNA Polymerase (Thermo Fisher Scientific Biosciences GmbH, St. Leon-Rot, Germany). Real-Time PCR was performed using a 7500 Real-Time PCR System (Applied Biosystems*,* Foster City, CA, USA). Primers were synthesized by Metabion International AG (Martinsried, Germany). PCR products were purified using the MSB^®^ Spin PCRapace Kit (STRATEC Biomedical AG, Berlin, Germany) and sequenced on both strands through sequencing service (Eurofins MWG GmbH, Ebersberg, Germany). Genotyping of the *vcg* and 16S rRNA genes was performed by conventional PCR amplification and Real-Time PCR, respectively [13,14]. In addition, all *V. vulnificus* isolates were tested for the presence of the gene *nanA* belonging to the sialic acid catabolism cluster and the gene *manIIA* of a mannitol fermentation operon by PCR amplification using published primer pairs [35,36]. The presence of genomic region XII was determined by amplification of the 5′ flanking region together with parts of the first gene VVA1613 of region XII encoding a chondroitinase AC lyase (VVA1612F and VVA1613R, 2257 bp). A second PCR assay, targeting the flanking regions, generates an amplicon only when region XII is completely absent (VVA1612bF and VVA1637R, 1200 bp). All strains were further tested for presence of VVA1634 of region XII, encoding an arylsulfatase A (VVA1634 F and VVA1634R, 1364 bp). Strains that contained region XII but were negative for VVA1634 were further tested with an additional primer pair (VVA1633a_F and VVA1635c_R, 2483 bp) designed based on published genome sequences (MO6-24/O, CMCP6 and YJ016) to amplify the whole arylsulfatase A gene. Generated amplicons of two strains (VN-10 and VN-16, characterized in Bier *et al.* [11]) were selected for further sequence analysis using a set of eleven primers. The sequences of the arylsulfatase A gene of VN-0010 (accession number LN879390) and VN-0016 (accession number LN879391) have been deposited in GenBank. All primer pairs, target genes, corresponding annealing temperatures, and amplicon sizes are listed in Supplementary Table S4.

### 2.5. Serum Resistance

A colorimetric assay that allows high-throughput screening of bacterial resistance to human serum was employed as previously described [11]. Experiments were performed in triplicate. Isolates that showed growth in the presence of 60%–80% human serum were classified as serum resistant. Isolates that only grew in the presence of 20%–40% or 0%–10% human serum were classified as intermediate resistant or susceptible, respectively.

### 2.6. Statistical Analyses

Descriptive statistics were used to compare the distribution of virulence-associated traits and genotypes among *V. vulnificus* isolates from the North Sea and Baltic Sea. Chi-square test for independence was applied with two-by-two contingency tables to test if observed differences regarding the geographical origin were statistically significant (*p*-values ≤ 0.05).

## 3. Results and Discussion

### 3.1. Multilocus Sequence Typing

Multilocus sequence typing (MLST) was performed on ten housekeeping genes according to Bisharat *et al.* [8,9] and revealed high genetic diversity among environmental *V. vulnificus* isolates from German coastal waters. Among the 101 environmental *V. vulnificus* isolates examined in this study, a total of 74 different sequence types (STs) were identified, including 65 new STs with 127 newly identified allele sequences. The majority of STs (73%) were present only once in the strain collection. Fourteen STs (19%) were represented by two isolates and only six STs (ST128, ST219, ST226, ST244, ST269 and ST268) by three to four isolates. None of the STs represented by more than one isolate showed geographical dispersion over both investigated areas. The ratio between the number of different STs (Table 1) and the number of strains was comparable between the North Sea (0.76) and the Baltic Sea (0.71), indicating a similar degree of genetic diversity.

**Table 1:** Distribution of MLST clusters and virulence-associated traits and genotypes among *V. vulnificus* isolates from the Baltic Sea and the North Sea.

Geographical Region (Number of Strains)	MLST Cluster (%)			*vcg-*Type (%)		16S rRNA-Type (%)			*nanA* (%)	Region XII (%)	Mannitol Fermentation (%)	Serum Resistance (%)			Risk Group (%) *^a^*		No. of Different Virulence Profiles (N)	No. of Strains (N)
–	I	IIA	IIB	C	E	A	AB	B	–	–	–	R	I	S	1	2	–	–
Total (*n* = 101)	79	6	15	6	94	79	14	7	47	37	40	79	14	7	42	59	17	74
Baltic Sea (*n* = 51)	71	0	29	0	100	71	27	2	24	35	14	76	18	6	61	39	8	36
North Sea (*n* = 50)	88	12	0	12	88	88	0	12	70	38	66	82	10	8	22	78	14	38

MLST, multilocus sequence typing; R, resistant; I, intermediate resistant; S, susceptible; ST, sequence type. *^a^* Risk Group 2 comprising strains with two or more pathogenicity markers, Risk Group 1 comprising strains without or with one pathogenicity marker.

The high genetic diversity is also demonstrated by the high number of different STs found among isolates originating from the same sampling site on the same date. For example, five different STs were observed among six strains recovered on the same date at a beach in Binz (Sampling Site 26).

**Figure 2:**
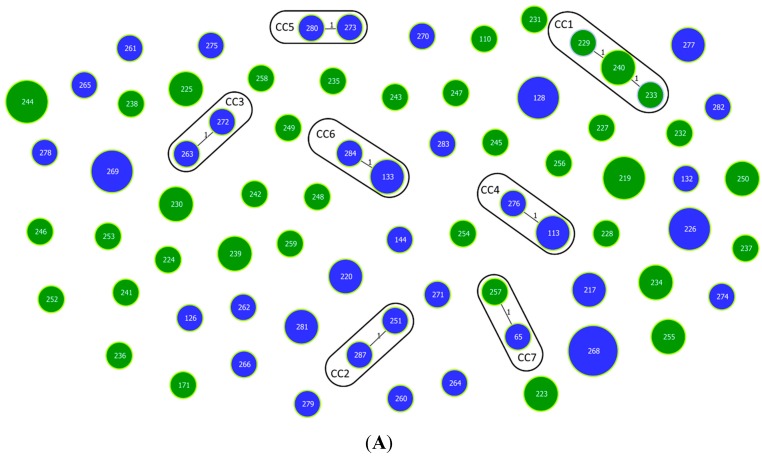

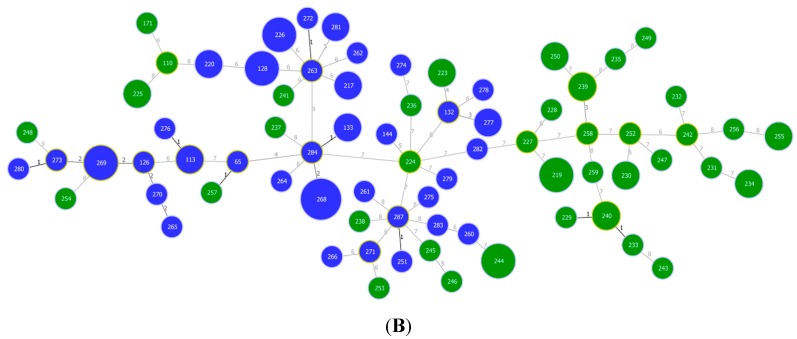
Population structure of *V. vulnificus* biotype 1 isolates from the North Sea and Baltic Sea obtained by goeBURST analysis based on MLST allelic profiles. Each sequence type (ST) is displayed as a circle with a size proportional to the number of isolates by which it is represented. The different colors indicate the geographical origin: North Sea (green) and Baltic Sea (blue). Single locus variants (SLVs) are connected via black lines. Light green halos around the circles indicate the respective founder of the group. (**A**) Population snapshot based on MLST allelic profiles. Clonal complexes (CC1–CC7) formed at the SLV level are highlighted by black edging; (**B**) Full Minimum Spanning Tree based on MLST allelic profiles. The number of different alleles between two STs is shown next to the connection lines.

Investigation of clonal relationships using the goeBURST algorithm revealed seven clonal complexes defined at the single locus variant (SLV) level, including one triplet (ST229-ST233-ST240 with ST240 as the predicted founder) and six doublets (ST251-ST287, ST272-ST263, ST113-ST276, ST273-ST280, ST133-ST284 and ST65-257) (Figure 2A). When double and triple locus variants (DLV and TLV) were considered in the analysis, two previously observed doublets were expanded (ST273-ST280-ST269-ST126-ST270-ST265 and ST133-ST284-ST268) and two additional doublets (ST239-ST258 and ST132-ST277) were assigned (Figure 2B). The remaining 50 STs were singletons that differ in four to nine loci from other STs. The Minimum Spanning Tree drawn using an extension of the goeBURST rules shows that most of these singletons (62%) differ in six or seven loci from other STs in the tree (Figure 2B). Of the nine clonal complexes identified at the TLV level, only one doublet is associated with isolates from both seas (ST65-257, CC7), whereas all other groups consisted of strains recovered from either the North Sea or the Baltic Sea (Figure 2B).

**Figure 3:**
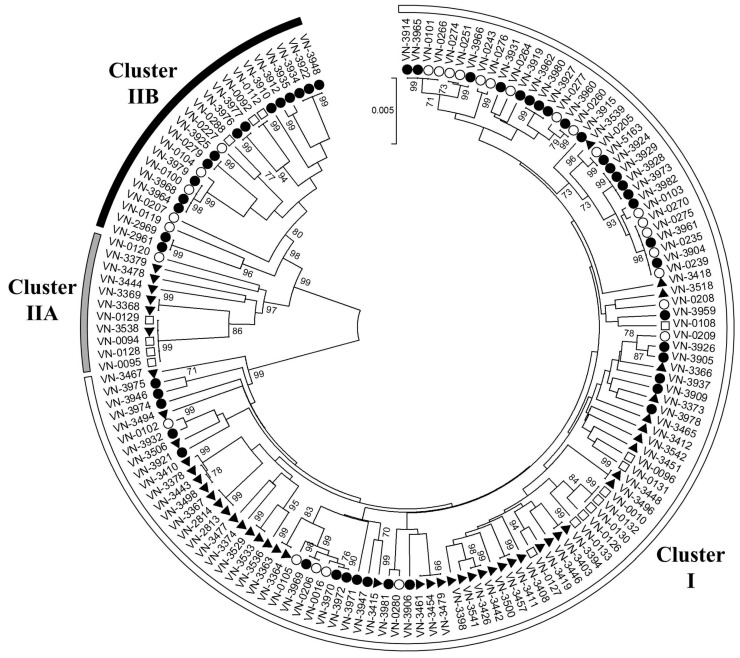
Population structure of *V. vulnificus* biotype 1 isolates from the North Sea (▲) and Baltic Sea (●) based on concatenated MLST sequences of ten housekeeping genes. Bootstrap values above 70% are shown next to the branches. Semicircles around the tree highlight the association of strains to MLST cluster I (white), IIA (grey), and IIB (black). Sequences from clinical (□) and environmental (○) Baltic Sea isolates from a previous study [11] were included for comparison.

As analysis of clonal relationships using allelic profiles does not consider the degree of heterogeneity at the nucleotide sequence level, an UPGMA tree was constructed based on the alignment of concatenated allele sequences to further evaluate genetic relationships among the strains (Figure 3).

Isolates from the Baltic Sea region characterized in our previous study [11] were included to increase the dataset and to identify strains with a close relationship to clinical strains. All isolates were divided into two major clusters I and II, as previously reported for MLST of *V. vulnificus* [8,9,10,11,16,37]. The majority of environmental isolates from the Baltic Sea (71%) and the North Sea (88%) investigated in this study fall into the “environmental” cluster I (Table 1). Twelve per cent of the North Sea isolates belonged to the “clinical” cluster II [8,9,10,11,16,37]. The proportion of cluster II isolates was more than twice as high among isolates from the Baltic Sea (29%), which was statistically significant (χ^2^ = 6.941, degrees of freedom (*df*) = 1, *p* < 0.05). Additionally, further separation of cluster II into two branches A and B was observed, concordantly with our previous analysis of clinical and environmental isolates from the Baltic Sea region [11]. Interestingly, all cluster II isolates of the North Sea fall into branch A, while those of the Baltic Sea were found in branch B (Figure 3). Although in this study, cluster IIA strains were exclusively obtained from the North Sea, our previous investigations had shown their existence in the Baltic Sea [11].

Cluster IIB strains were obtained from sampling sites distributed along the whole Baltic Sea coast (Figure 1) and accounted for a significant proportion of the *V. vulnificus* Baltic Sea isolates (29%). While none of the North Sea isolates investigated in this study fell into this branch, a cluster IIB strain originating from the Netherlands (strain no. 478867 and ST138 isolated in 1998 from fish/eel) is stored in the PubMLST database, indicating that cluster IIB also occurs in the North Sea. All cluster IIB strains listed in the PubMLST database (*n* = 19) were isolated in Europe. Most of them (*n* = 17) originate from the Baltic Sea region, thus indicating that cluster IIB strains represent a characteristic part of the *V. vulnificus* population in the Baltic Sea.

Interestingly, some environmental isolates are highly related to clinical strains and may represent a public health risk. For example, the North Sea isolate VN-3538 within cluster IIA shows the same sequence type found in four clinical strains (VN-0094, VN-0095, VN-0128 and VN-0129) from the Baltic Sea region. These five strains cluster together with two additional isolates from the North Sea (VN-3369 and VN-3368) indicating a close relationship. Another subcluster is found within cluster IIB. The Baltic Sea isolates VN-03977 and VN-03976 form a well supported group together with the clinical isolates VN-0092 and VN-0112. Within cluster I, one clinical isolate (VN-0127) and one North Sea isolate (VN-3419) form a group.

A dendrogram with a comparable topology regarding the two major clusters and subclusters was obtained using the concatenated sequences of only three loci (*gyrB*, *dtdS* and *pyrC*) (Supplementary Figure S1). Consequently, MLST analysis based on these three loci seems to be sufficient for rapid identification of cluster I and II, respectively.

### 3.2. Distribution of Virulence-Associated Traits and Genotypes in MLST Clusters

**Figure 4:**
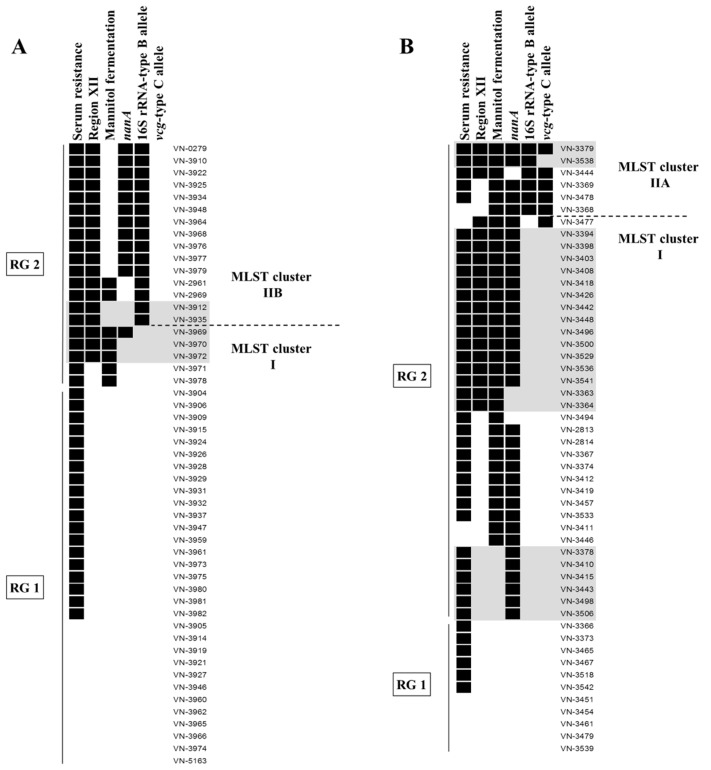
Combined results of MLST analysis and the investigation of virulence-associated traits and genotypes among *V. vulnificus* isolates from the Baltic Sea (**A**) and the North Sea (**B**). Presence of a pathogenicity marker is indicated by a black box. Strains rated as resistant (growth in 60%–80% human serum) are displayed as positive for serum resistance. Virulence profiles that have already been found in clinical isolates from the Baltic Sea region [11] are highlighted in grey. Risk Group 2 (RG2) comprises strains with two or more pathogenicity markers, while strains without or with one pathogenicity marker were assigned to Risk Group 1 (RG1) (see text). The figure was created using BioNumerics v7.5 (Applied Maths, Sint-Martens-Latem, Belgium).

All 101 *V. vulnificus* isolates were classified as biotype 1 based on multiplex PCR analysis and biochemical characteristics (positive indole reaction, negative for sorbitol fermentation). The isolates were examined for the ability to grow in human serum and to ferment mannitol (phenotypically and genotypically), as well as for the presence of the virulence-associated *nanA* gene and genomic region XII. In addition, all strains were typed based on the *vcg* and 16S rRNA genes to differentiate clinical associated C-type strains (16S rRNA-type B, *vcg*-type C) from environmental associated E-type strains (16S rRNA-type A, *vcg*-type E). All results are displayed in detail in supplementary Table S5. In addition, Figure 4 illustrates the distribution of virulence-associated traits and genotypes among the different MLST clusters and geographical areas.

The investigations revealed cluster-specific distributions of the 16S rRNA and *vcg* genotypes in the strain collection. MLST cluster I (*n* = 80) comprised all A/E-type isolates observed in this study and one A/C-type isolate (VN-3477). All cluster IIA strains (*n* = 6) were 16S rRNA-type B and *vcg*-type C, with the exception of one B/E-type isolate, which is in accordance with previous reports [10,11]. All strains of MLST cluster IIB (*n* = 15) were of the *vcgE*-type and characterized by possessing the 16S rRNA-type B allele, either alone (type B, 7%) or in combination with the type A allele (type AB, 93%). The presence of both 16S rRNA alleles in one strain has already been described before, as multiple copies of rRNA loci exist per genome [10,11,14]. In all AB-type isolates of this study, amplification of the type B allele reached the threshold at an earlier cycle compared to that of the type A allele. Thus, the type B allele seems to be present in a higher copy number in these isolates. The 16S rRNA-type B is regarded as an indicator for strain virulence, as the majority of clinical isolates were found to be type B (76%) [14]. In addition, a considerable proportion of clinical isolates has been described to possess both alleles (15%) [14], and 16S rRNA-type B as well as type AB strains are associated with a higher ability to cause lethal systemic infections in mice [15].

Overall, these results confirm correlations found in other studies [10,11] between MLST clusters and genotypes of the *vcg* and 16S rRNA genes, with type B/C being indicative for MLST cluster IIA and type AB/E or B/E being indicative for MLST cluster IIB. However, exceptions regarding the *vcg*-type observed in this and other studies [10] imply that these correlations are not absolute.

In our previous study [11] all cluster IIA isolates were of the B/C-type, resistant to human serum, and were positive for *nanA*, region XII, and mannitol fermentation. In contrast, cluster IIA strains of this study showed a higher variability of virulence-associated traits. All cluster IIA strains were able to ferment mannitol. This characteristic was also observed in 40% and 13% of cluster I and IIB isolates, respectively. The majority of isolates belonging to cluster IIA (83%) and IIB (73%) were positive for the gene *nanA*, while this was only the case for 39% of cluster I isolates. All cluster IIB and the majority of cluster IIA (83%) and cluster I (75%) isolates showed growth in the presence of 60%–80% human serum. 100% of cluster IIB and 50% of cluster IIA isolates contained region XII. 24% of cluster I isolates also showed presence of region XII, whereby cluster-specific differences were observed regarding the arylsulfatase A gene VVA1634. Overall, MLST cluster II showed a higher prevalence of the clinical associated region XII, *nanA* and the mannitol fermentation operon, confirming observations of other studies [10,11,36].

The 33-kb genomic region XII has been implicated to contribute to virulence of *V. vulnificus*, as this region has predominantly been found in clinical isolates and in strains possessing the clinical genotype (16S rRNA-type B and *vcg*-type C) [10,11]. Region XII encompasses 22 open reading frames, including two chondroitinases and an arylsulfatase A gene cluster that may contribute to pathogenesis either directly or by simply mediating a selective advantage within the human host [10,38]. For detection and characterization of region XII, three PCR assays were conducted. In one PCR we targeted the 5′ flanking sequence and the first gene of region XII encoding a chondroitinase AC lyase (VVA1613). Amplification of this region is in complete accordance with the presence of region XII [10,11]. A second PCR, targeting the 5′ and 3′ flanking sequences confirmed absence of the whole region XII in all VVA1613-negative strains.

A third PCR assay targeted the gene VVA1634 of region XII encoding an arylsulfatase A. As previously observed [11], this gene could only be detected in region XII-positive strains of MLST cluster II with the used primers. In contrast, no amplicon was generated in region XII containing strains of MLST cluster I (*n* = 17). To elucidate if VVA1634 is absent or if possible single nucleotide polymorphisms (SNPs) prevent amplification in these strains, we designed primers to amplify the whole arylsulfatase A gene. In 14 cases a product with the expected size of 2.5 kb could be generated, indicating that the gene is present. Sequencing of the amplicon in selected strains and comparison with the two genome sequenced reference strains of MLST cluster II (CMCP6 and MO6-24/O) revealed cluster-specific gene polymorphisms, including both primer binding sites of VVA1634F and VVA1634R. The absence of the gene in the remaining three strains cannot be excluded, however, it seems likely that more SNPs may have prevented amplification in these strains. In total, 71 SNPs were found in VVA1634 between strains of MLST cluster I and II resulting in eighteen amino acid exchanges. These cluster specific variations should be considered when examining the role of this arylsulfatase A for pathogenesis of *V. vulnificus.*

### 3.3. Distribution of Virulence-Associated Traits and Genotypes among Isolates from the North Sea and the Baltic Sea

Data analysis revealed distinct differences in the distribution of virulence-associated traits and genotypes among the North Sea and Baltic Sea isolates (Figure 4). *Vcg*-type C strains were exclusively found among the North Sea isolates, and the 16S rRNA-type B allele was observed in a greater proportion of Baltic Sea isolates (29% type AB or B) compared to the North Sea isolates (12%) (Table 1). These observations match the geographical distribution pattern of MLST cluster IIA and IIB isolates described above.

The majority of the North Sea isolates were positive for the *nanA* gene (70%) and for mannitol fermentation (66%) while this was only the case in 24% and 14% of the Baltic Sea isolates, respectively (Table 1). These differences proved to be statistically significant (χ^2^ = 25.54; *df* = 1, *p* < 0.001 for *nanA* and χ^2^ = 32.72; *df* = 1, *p* < 0.001 for mannitol fermentation). Region XII was evenly distributed in 35% and 38% of the North Sea and Baltic Sea isolates, respectively. In addition, the proportions of isolates showing intermediate resistance and resistance to human serum were similar among the North Sea and Baltic Sea isolates (Table 1).

In this study, a total of seventeen different combinations (virulence profiles, VPs) of the six investigated virulence-associated traits and genotypes were identified among the 101 environmental *V. vulnificus* isolates (Table 2). Six VPs were already observed in clinical isolates [11]. The North Sea isolates showed a higher variability regarding the different combinations of virulence-associated traits, with fourteen different VPs compared to eight VPs found among the Baltic Sea isolates (Table 1). Nine VPs were only found among the North Sea isolates, three VPs were specifically detected among the Baltic Sea isolates, and five VPs were identified in isolates from both seas (Table 2). Isolates sharing the same virulence profile belonged to the same MLST cluster (Table 2). In total, the observed differences in the geographical distribution of virulence-associated traits and genes, as well as of distinct VPs show that the populations of the North Sea and the Baltic Sea vary widely.

### 3.4. Assessment of the Pathogenicity Potential of Environmental Strains

Several MLST analyses conducted on *V. vulnificus* demonstrated that the majority of strains of MLST cluster II are of clinical origin [8,9,10,11,16,37]. This cluster is also highly correlated with the clinical associated alleles of the *vcg* and 16S rRNA genes (*vcg*-type C, 16S rRNA-type B) that are often used to predict strain virulence [12,13,39]. In addition, the majority of cluster II strains are positive for the *nanA* gene, region XII, and mannitol fermentation which are traits associated with clinical origin [10,11,16,36]. Environmental isolates of cluster II are therefore assumed to have a high potential to cause infection and were categorized into Risk Group 2 in this study (Table 2).

**Table 2:** Virulence profiles and corresponding number of *V. vulnificus* strains with respect to geographical origin and MLST cluster.

Risk Group *^a^*	Virulence Profile	No. of Isolates (Geographical Origin)	MLST Cluster
Risk Group 1	–	17 (12 BS, 5 NS)	I
	Res	25 (19 BS, 6 NS)	I
Risk Group 2	Man-*nanA*	2 (2 NS)	I
	Region XII-Man-*nanA*-vcgC	1 (1 NS)	I
	Res-Man	3 (2 BS, 1 NS)	I
	Res-Man-*nanA*	8 (8 NS)	I
	Res-*nanA* *^b^*	6 (6 NS *^c^*)	I
	Res-Region XII-Man *^b^*	4 (2 BS, 2 NS)	I
	Res-Region XII-Man-*nanA* *^b^*	14 (1 BS, 13 NS)	I
	Man-*nanA*-16S_B-vcgC	1 (1 NS)	IIA
	Res-Man-*nanA*-16S_B-vcgC	2 (2 NS)	IIA
	Res-Region XII-16S_B *^b^*	2 (2 BS)	IIB
	Res-Region XII-Man-16S_B	2 (2 BS)	IIB
	Res-Region XII-Man-16S_B-vcgC	1 (1 NS)	IIA
	Res-Region XII-Man-*nanA*-16S_B *^b^*	1 (1 NS *^c^*)	IIA
	Res-Region XII-Man-*nanA*-16S_B-vcgC *^b^*	1 (1 NS *^c^*)	IIA
	Res-Region XII-*nanA*-16S_B	11 (11 BS)	IIB

MLST, multilocus sequence typing; Res, growth in 60%–80% human serum; Man, mannitol fermentation; 16S_B, presence of 16S rRNA-type B allele in type B or type AB; BS, Baltic Sea; NS, North Sea. *^a^* Risk Group 2 comprising strains with two or more pathogenicity markers, Risk Group 1 comprising strains without or with one pathogenicity marker. *^b^* profile already found in clinical isolates from the Baltic Sea region [11]. *^c^* profile exclusively found in the North Sea in this study, but previously observed among clinical or environmental isolates from the Baltic Sea region [11].

In our previous study, we observed that the majority of clinical biotype 1 isolates from cases in the Baltic Sea region (59%) belonged to the “environmental” MLST cluster I and were of the A/E-genotype. However, these clinical isolates were characterized by the presence of the *nanA* gene, region XII, or the ability to ferment mannitol. Therefore, environmental strains of MLST cluster I possessing at least one of these pathogenicity markers were also assigned to Risk Group 2. Isolates that were negative for all tested virulence-associated traits or that showed only serum resistance were merged in Risk Group 1, as they possess the lowest probability to cause human infection.

Among the Baltic Sea isolates, the majority of strains belonged to Risk Group 1 (61%). In contrast, the majority of the North Sea isolates were assigned to Risk Group 2 (78%). Only a small proportion of strains (22%) was likely to be less pathogenic as no or only one single pathogenicity marker was present (Table 1, Figure 4). Remarkably, this significant difference (χ^2^ = 18.947, *df* = 1, *p* < 0.001) is in contradiction with the observed prevalence of infection in both geographical areas. All cases of *V. vulnificus* infection in Germany were documented for the Baltic Sea region, while no cases have been reported from the North Sea coast so far. One explanation for this discrepancy could be the relatively rare occurrence and lower abundance of *V. vulnificus* in the North Sea, where the presence of *V. vulnificus* is restricted to low-salinity environments in the river estuaries of Weser and Ems [18,23]. In contrast, *V. vulnificus* has repeatedly been found at bathing sites along the whole German Baltic Sea coast of Mecklenburg-Western Pomerania, where it represents the most abundant potentially pathogenic *Vibrio* species together with *V. cholerae* non-O1/non-O139 [23,40].

Apart from differences in occurrence and abundance of potential pathogenic *V. vulnificus* in the two regions, more aspects have to be taken into account when reasons for the lack of cases in the North Sea region are sought for. Missing information on infections may be a result of underreporting and lack of public perception for this pathogen in the North Sea region. Furthermore, data on recreational activities in relation to water and weather conditions (temperature, salinity, *etc.*) are of great relevance, as infections are the result of exposition to the pathogen. In this study, only molecular characteristics of *V. vulnificus* isolates from the two areas were investigated.

The results of our study reveal that a high proportion of North Sea isolates could be of clinical significance (78%). This finding is of concern, as an increase in the occurrence of *V. vulnificus* in the North Sea is expected due to climate change. Multiple linear regression models based on the presence and abundance of *V. vulnificus* at the German North Sea coast between 2009 and 2011 estimate an increase of up to 30% for the occurrence of *V. vulnificus* by the end of 2099 [23]. Furthermore, an expansion of low-salinity habitats after heavy rainfall events would support a further dissemination of *V. vulnificus* along the German North Sea coast [23].

## 4. Conclusions

The aim of this study was to compare MLST profiles and virulence-associated traits of *V. vulnificus* strains isolated from the German North Sea and Baltic Sea coasts and to assess their pathogenicity potential. Climate change will favor growth conditions of mesophilic vibrios in the next decades in these two seas and *V. vulnificus* is expected to benefit greatly from rising sea surface temperatures [20,21]. As the two German coastlines are popular recreational areas visited by millions of tourists every year, a risk assessment for strains of this potentially dangerous species is heavily in demand. Isolates of *V. vulnificus* from the German North Sea coastline have not been investigated in detail before and studies addressing the pathogenicity potential of *V. vulnificus* from the Baltic Sea are scarce [11]. In this study a high genetic diversity was observed by MLST among North Sea and Baltic Sea isolates. Combination of MLST data and presence of virulence-associated traits suggested grouping of strains unlikely to cause human infection in Risk Group 1. Analysis of the *V. vulnificus* populations of the two areas revealed that the proportion of strains with two or more pathogenicity markers (Risk Group 2) was higher in the North Sea than in the Baltic Sea. Although the occurrence of *V. vulnificus* in the North Sea is lower than in the Baltic Sea and restricted to the river estuaries [18,23], this study clearly demonstrates that increased awareness for this pathogen by both the public health sector and local medical staff is required for the North Sea region.

## Supplementary Files

Supplementary File 1
