# Supplementary material for: Virulence Profiles of Vibrio vulnificus in German Coastal Waters, a Comparison of North Sea and Baltic Sea Isolates
Source: Int J Environ Res Public Health. 2015 Dec 15;12(12):15943–59. doi: 10.3390/ijerph121215031 (PMC4690967; doi:10.3390/ijerph121215031)
Supplement: Supplementary File 1 [file ijerph-12-15031-s001.pdf]

Virulence Profiles of *Vibrio vulnificus* in German Coastal Waters, a Comparison of North Sea and Baltic Sea Isolates

Table S1. Sampling sites, classification and bathing water quality.

Geographical Region	Sampling Site No.	Sampling Site	No. of Strains	Classification (Coastal Waters) [a]	Bathing Water Quality [b]
North Sea	1	Borkum	1	euhaline open coastal waters	excellent
	2	Dyksterhusen	3	mesohaline inner coastal waters	good
	3	Jemgum	1	mesohaline inner coastal waters	no designated beach
	4	Burhave	8	mesohaline inner coastal waters	excellent
	5	Dedesdorf	9	mesohaline inner coastal waters	no designated beach
	6	Kleinensiel	2	mesohaline inner coastal waters	no designated beach
	7	Bremerhaven	16	mesohaline inner coastal waters	no designated beach
	8	Wremen	5	mesohaline inner coastal waters	excellent
	9	Altenbruch	3	mesohaline inner coastal waters	excellent
		k.A.	2		
Baltic Sea	10	Mönkeberg	1	mesohaline inner coastal waters	Excellent
	11	Kiel-Dietrichsdorf	1	mesohaline inner coastal waters	no designated beach
	12	Wohlenberger Wiek	2	mesohaline inner coastal waters	good
	13	Kühlungsborn	1	mesohaline open coastal waters	excellent
	14	Warnemünde	6	mesohaline open coastal waters	excellent
	15	Darss-Zingster Bodden chain, station 9	2	mesohaline inner coastal waters	no designated beach
	16	Darss-Zingster Bodden chain, station 8	2	mesohaline inner coastal waters	no designated beach
	17	Darss-Zingster Bodden chain, station 7	4	mesohaline inner coastal waters	no designated beach
	18	Greifswalder Bodden, station 5	2	mesohaline inner coastal waters	no designated beach
	19	Greifswalder Bodden, station 4	3	mesohaline inner coastal waters	no designated beach
	20	Greifswalder Bodden, station 3	5	mesohaline inner coastal waters	no designated beach

Table S1. *Cont.*

Geographical Region	Sampling Site No.	Sampling Site	No. of Strains	Classification (Coastal Waters) [a]	Bathing Water Quality [b]
Baltic Sea	21	Greifswalder Bodden, station 2	4	mesohaline inner coastal waters	no designated beach
	22	Lubmin	4	mesohaline inner coastal waters	excellent
	23	Karlshagen	3	mesohaline open coastal waters	excellent
	24	Trassenheide	1	mesohaline open coastal waters	excellent
	25	Greifswalder Bodden, station 1	2	mesohaline inner coastal waters	no designated beach
	26	Binz	7	mesohaline open coastal waters	excellent
	27	Rügen	1	mesohaline inner coastal waters	no designated beach

[a] according to the European Water Framework Directive 2000/60/EC. [b] according to the requirements of the European Bathing Water Directive 2006/7/EC; results from 2011.

Table S2. Detailed sampling information of *V. vulnificus* isolates examined in this study.

Strain ID	Origin	Sampling Site No. [a]	Sampling Site Name	Seawater Temperature (°C)	Seawater Salinity (psu)	Sampling Date
VN-0279	B-sw	12	Wohlenberger Wiek	15.5	13	06.09.2011
VN-2813	N-sw	-	n.s.	n.s.	n.s.	16.05.2011
VN-2814	N-sw	-	n.s.	n.s.	n.s.	16.05.2011
VN-2961	B-sw	11	Kiel-Dietrichsdorf	18	n.s.	25.07.2011
VN-2969	B-sw	10	Mönkeberg	17.4	n.s.	25.07.2011
VN-3363	N-sd	4	Burhave	25.6	21.7	12.07.2010
VN-3364	N-sd	4	Burhave	25.6	21.7	12.07.2010
VN-3366	N-sd	4	Burhave	25.6	21.7	12.07.2010
VN-3367	N-sw	7	Bremerhaven	24.8	17.0	12.07.2010
VN-3368	N-sw	7	Bremerhaven	24.8	17.0	12.07.2010
VN-3369	N-sw	7	Bremerhaven	24.8	17.0	12.07.2010
VN-3373	N-sd	5	Dedesdorf	20.4	8.2	12.07.2010
VN-3374	N-sd	5	Dedesdorf	20.4	8.2	12.07.2010
VN-3378	N-sw	2	Dyksterhusen	20.4	20.5	20.07.2010

Table S2. *Cont.*

Strain ID	Origin	Sampling Site No. [a]	Sampling Site Name	Seawater Temperature (°C)	Seawater Salinity (psu)	Sampling Date
VN-3379	N-sw	2	Dyksterhusen	20.4	20.5	20.07.2010
VN-3394	N-sd	8	Wremen	21.7	22.5	10.08.2010
VN-3398	N-sw	8	Wremen	21.7	22.5	10.08.2010
VN-3403	N-sw	8	Wremen	21.7	22.5	10.08.2010
VN-3408	N-sw	2	Dyksterhusen	19	14.2	17.08.2010
VN-3410	N-sw	4	Burhave	18.7	25.1	18.08.2010
VN-3411	N-sd	4	Burhave	18.7	25.1	18.08.2010
VN-3412	N-sw	7	Bremerhaven	20.5	19.3	18.08.2010
VN-3415	N-sw	7	Bremerhaven	20.5	19.3	18.08.2010
VN-3418	N-sd	7	Bremerhaven	20.5	19.3	18.08.2010
VN-3419	N-sw	5	Dedesdorf	20.7	6.2	18.08.2010
VN-3426	N-sd	5	Dedesdorf	20.7	6.2	18.08.2010
VN-3442	N-sd	1	Borkum	16	29.8	24.08.2010
VN-3443	N-sd	4	Burhave	15	11.8	07.09.2010
VN-3444	N-sd	4	Burhave	15	11.8	07.09.2010
VN-3446	N-sd	7	Bremerhaven	15	11.8	07.09.2010
VN-3448	N-sw	7	Bremerhaven	17.5	14.6	07.09.2010
VN-3451	N-sw	7	Bremerhaven	17.5	14.6	07.09.2010
VN-3454	N-sd	7	Bremerhaven	17.5	14.6	07.09.2010
VN-3457	N-sd	7	Bremerhaven	17.5	14.6	07.09.2010
VN-3461	N-sw	5	Dedesdorf	17.7	1.9	07.09.2010
VN-3465	N-sd	5	Dedesdorf	17.7	1.9	07.09.2010
VN-3467	N-sd	5	Dedesdorf	17.7	1.9	07.09.2010
VN-3477	N-sw	7	Bremerhaven	14.1	14.9	05.10.2010
VN-3478	N-sd	7	Bremerhaven	14.1	14.9	05.10.2010
VN-3479	N-sd	5	Dedesdorf	15.6	5.3	05.10.2010

Table S2. *Cont.*

Strain ID	Origin	Sampling Site No. [a]	Sampling Site Name	Seawater Temperature (°C)	Seawater Salinity (psu)	Sampling Date
VN-3494	N-sw	7	Bremerhaven	17.5	14.6	07.09.2010
VN-3496	N-sw	7	Bremerhaven	17.5	14.6	07.09.2010
VN-3498	N-sd	4	Burhave	15	11.8	07.09.2010
VN-3500	N-sd	7	Bremerhaven	20.5	12.4	11.07.2011
VN-3506	N-sw	5	Dedesdorf	17.7	1.9	07.09.2010
VN-3518	N-sw	9	Altenbruch	n.s.	n.s.	12.06.2012
VN-3529	N-sw	6	Kleinensiel	n.s.	n.s.	02.08.2012
VN-3533	N-sw	8	Wremen	n.s.	n.s.	07.08.2012
VN-3536	N-sw	9	Altenbruch	n.s.	n.s.	07.08.2012
VN-3538	N-sw	6	Kleinensiel	n.s.	n.s.	13.08.2012
VN-3539	N-sw	3	Jemgum	n.s.	n.s.	14.08.2012
VN-3541	N-sw	8	Wremen	n.s.	n.s.	21.08.2012
VN-3542	N-sw	9	Altenbruch	n.s.	n.s.	06.09.2012
VN-3904	B-sd	19	Greifswalder Bodden, station 4	19.0	6.6	05.07.2011
VN-3905	B-sd	19	Greifswalder Bodden, station 4	19.0	6.6	05.07.2011
VN-3906	B-sd	18	Greifswalder Bodden, station 5	19.3	7.2	05.07.2011
VN-3909	B-sd	17	Darss-Zingster Bodden chain, station 7	19.2	7.9	05.07.2011
VN-3910	B-sd	17	Darss-Zingster Bodden chain, station 7	19.2	7.9	05.07.2011
VN-3912	B-sd	16	Darss-Zingster Bodden chain, station 8	19.0	7.1	05.07.2011

VN-3914	B-sd	15	Darss-Zingster Bodden chain, station 9	18.4	7.3	05.07.2011
VN-3915	B-sd	15	Darss-Zingster Bodden chain, station 9	18.4	7.3	05.07.2011
VN-3919	B-sd	25	Greifswalder Bodden, station 1	18.4	6.7	06.07.2011
VN-3921	B-sd	21	Greifswalder Bodden, station 2	18.9	6.6	06.07.2011
VN-3922	B-sw	20	Greifswalder Bodden, station 3	19.1	6.4	06.07.2011
VN-3924	B-sd	20	Greifswalder Bodden, station 3	19.1	6.4	06.07.2011
VN-3925	B-sd	12	Wohlenberger Wiek	18.9	10.7	01.08.2011

Table S2. *Cont.*

Strain ID	Origin	Sampling Site No. [a]	Sampling Site Name	Seawater Temperature (°C)	Seawater Salinity (psu)	Sampling Date
VN-3926	B-sd	25	Greifswalder Bodden, station 1	18,2	7,3	02.08.2011
VN-3927	B-sd	21	Greifswalder Bodden, station 2	19,5	6,2	02.08.2011
VN-3928	B-sw	20	Greifswalder Bodden, station 3	18,9	6,2	02.08.2011
VN-3929	B-sd	20	Greifswalder Bodden, station 3	18,9	5,9	02.08.2011
VN-3931	B-sd	20	Greifswalder Bodden, station 3	18,9	5,9	02.08.2011
VN-3932	B-sd	19	Greifswalder Bodden, station 4	19,7	6,3	02.08.2011
VN-3934	B-sd	17	Darss-Zingster Bodden chain, station 7	19,7	5,6	03.08.2011
VN-3935	B-sd	16	Darss-Zingster Bodden chain, station 7	19,7	5,6	03.08.2011
VN-3937	B-sd	16	Darss-Zingster Bodden chain, station 8	20,7	3	03.08.2011
VN-3946	B-sd	21	Greifswalder Bodden, station 2	13,4	6,7	11.10.2011
VN-3947	B-sd	21	Greifswalder Bodden, station 2	13,4	6,7	11.10.2011
VN-3948	B-sd	18	Greifswalder Bodden, station 5	12,6	7,6	11.10.2011
VN-3959	B-sw	22	Lubmin	26	6,5	05.07.2010
VN-3960	B-sw	23	Karlshagen	21,1	5,4	19.07.2010
VN-3961	B-sw	23	Karlshagen	21,1	5,4	19.07.2010
VN-3962	B-sw	22	Lubmin	20	6,4	19.07.2010
VN-3964	B-sw	26	Binz	n.s.	6,7	23.07.2010
VN-3965	B-sw	26	Binz	n.s.	6,7	23.07.2010
VN-3966	B-sw	26	Binz	n.s.	6,7	23.07.2010
VN-3968	B-sw	26	Binz	n.s.	6,7	23.07.2010
VN-3969	B-sw	26	Binz	n.s.	6,7	23.07.2010
VN-3970	B-sw	22	Lubmin	n.s.	6,3	02.08.2010
VN-3971	B-sw	24	Trassenheide	20,9	6,6	02.08.2010
VN-3972	B-sw	23	Karlshagen	21,5	6,5	02.08.2010
VN-3973	B-sw	13	Kühlungsborn	20,2	8,3	02.08.2010

Table S2. *Cont.*

Strain ID	Origin	Sampling Site No. [a]	Sampling Site Name	Seawater Temperature (°C)	Seawater Salinity (psu)	Sampling Date
VN-3974	B-sw	14	Warnemünde	22,8	8	04.08.2010
VN-3975	B-sw	14	Warnemünde	22,8	8	04.08.2010
VN-3976	B-sw	14	Warnemünde	22,8	8	04.08.2010
VN-3977	B-sw	14	Warnemünde	22,8	8	04.08.2010
VN-3978	B-sw	14	Warnemünde	22,8	8	04.08.2010
VN-3979	B-sd	14	Warnemünde	22,8	8	04.08.2010
VN-3980	B-sw	26	Binz	21	6,6	09.08.2010
VN-3981	B-sw	22	Lubmin	5,4	19,5	11.07.2011
VN-3982	B-sw	26	Binz	n.s.	6,7	23.07.2010
VN-5163	B-sw	27	Rügen	n.s.	n.s.	12.06.2011

N, North Sea; B, Baltic Sea; sw, seawater; sd, sediment; n.s., not specified. [a] Sampling site numbers shown in Figure 1.

Table S3. Allelic profiles of the 101 *V. vulnificus* isolates tested (new STs/alleles are displayed in red).

Strain ID	MLST ST	MLST-Cluster	Clonal Complex (SLV-Level)	Clonal Complex (TLV-Level)	*glp*	*gyrB*	*mdh*	*metG*	*purM*	*dtdS*	*lysA*	*pntA*	*pyrC*	*tnaA*
VN-0279	217	IIB	Singleton	Singleton	12	39	42	29	57	27	41	77	13	11
VN-2813	219	I	Singleton	Singleton	70	57	2	9	8	97	4	6	78	73
VN-2814	219	I	Singleton	Singleton	70	57	2	9	8	97	4	6	78	73
VN-2961	220	IIB	Singleton	Singleton	4	58	12	13	12	24	92	1	13	19
VN-2969	220	IIB	Singleton	Singleton	4	58	12	13	12	24	92	1	13	19
VN-3363	223	I	Singleton	Singleton	49	40	44	24	8	107	6	65	5	50
VN-3364	223	I	Singleton	Singleton	49	40	44	24	8	107	6	65	5	50
VN-3366	224	I	Singleton	Singleton	47	14	2	7	8	19	33	15	5	74
VN-3367	219	I	Singleton	Singleton	70	57	2	9	8	97	4	6	78	73
VN-3368	225	IIA	Singleton	Singleton	44	1	37	59	25	98	106	1	23	7
VN-3369	225	IIA	Singleton	Singleton	44	1	37	59	25	98	106	1	23	7

Table S3. *Cont.*

Strain ID	MLST ST	MLST-Cluster	Clonal Complex (SLV-Level)	Clonal Complex (TLV-Level)	*glp*	*gyrB*	*mdh*	*metG*	*purM*	*dtdS*	*lysA*	*pntA*	*pyrC*	*tnaA*
VN-3373	227	I	Singleton	Singleton	7	23	2	22	8	22	4	66	56	18
VN-3374	228	I	Singleton	Singleton	71	12	2	41	8	22	69	67	56	73
VN-3378	229	I	1	2	19	61	73	60	65	100	96	80	12	76
VN-3379	171	IIA	Singleton	Singleton	55	1	4	5	30	66	42	10	37	53
VN-3394	230	I	Singleton	Singleton	72	60	2	24	9	22	12	13	84	18
VN-3398	231	I	Singleton	Singleton	19	42	13	12	8	22	104	21	7	49
VN-3403	230	I	Singleton	Singleton	72	60	2	24	9	22	12	13	84	18
VN-3408	232	I	Singleton	Singleton	24	23	70	12	9	35	12	68	80	30
VN-3410	233	I	1	2	19	61	2	60	65	100	96	80	11	76
VN-3411	234	I	Singleton	Singleton	19	42	13	62	40	82	12	13	64	15
VN-3412	235	I	Singleton	Singleton	7	11	11	22	59	94	65	69	12	51
VN-3415	236	I	Singleton	Singleton	8	62	2	2	13	19	75	9	5	18
VN-3418	237	I	Singleton	Singleton	73	15	16	7	9	35	91	13	33	9
VN-3419	238	I	Singleton	Singleton	9	23	11	24	8	96	56	11	12	9
VN-3426	239	I	Singleton	4	74	11	2	22	60	103	12	20	81	50
VN-3442	239	I	Singleton	4	74	11	2	22	60	103	12	20	81	50
VN-3443	240	I	1	2	19	61	73	60	65	100	96	80	11	76
VN-3444	241	IIA	Singleton	Singleton	35	63	3	25	25	105	114	36	43	77
VN-3446	242	I	Singleton	Singleton	75	42	2	12	9	22	12	70	82	78
VN-3448	250	I	Singleton	Singleton	26	2	2	3	62	110	12	20	84	83
VN-3451	243	I	Singleton	Singleton	76	64	2	60	8	106	98	13	83	79
VN-3454	244	I	Singleton	Singleton	10	28	13	7	8	107	26	71	15	80
VN-3457	234	I	Singleton	Singleton	19	42	13	62	40	82	12	13	64	15
VN-3461	244	I	Singleton	Singleton	10	28	13	7	8	107	26	71	15	80
VN-3465	245	I	Singleton	Singleton	47	14	17	17	8	44	18	21	4	9
VN-3467	246	I	Singleton	Singleton	18	19	17	17	8	5	95	21	4	26

Table S3. *Cont.*

Strain ID	MLST ST	MLST-Cluster	Clonal Complex (SLV-Level)	Clonal Complex (TLV-Level)	*glp*	*gyrB*	*mdh*	*metG*	*purM*	*dtdS*	*lysA*	*pntA*	*pyrC*	*tnaA*
VN-3477	247	I	Singleton	Singleton	71	23	2	12	61	22	113	7	26	62
VN-3478	248	IIA	Singleton	Singleton	77	65	71	63	23	108	41	72	59	81
VN-3479	244	I	Singleton	Singleton	10	28	13	7	8	107	26	71	15	80
VN-3494	249	I	Singleton	Singleton	71	66	44	64	59	109	100	69	11	82
VN-3496	250	I	Singleton	Singleton	26	2	2	3	62	110	12	20	84	83
VN-3498	240	I	1	2	19	61	73	60	65	100	96	80	11	76
VN-3500	252	I	Singleton	Singleton	78	67	2	22	9	22	12	73	26	10
VN-3506	253	I	Singleton	Singleton	52	28	11	23	8	34	60	21	5	52
VN-3518	254	I	Singleton	Singleton	79	16	75	2	9	111	17	18	85	45
VN-3529	255	I	Singleton	Singleton	80	23	9	12	63	111	64	74	9	84
VN-3533	255	I	Singleton	Singleton	80	23	9	12	63	111	64	74	9	84
VN-3536	256	I	Singleton	Singleton	81	43	9	12	63	22	93	74	86	85
VN-3538	110	IIA	Singleton	Singleton	38	1	12	31	27	23	36	1	48	47
VN-3539	257	I	7	9	13	14	15	40	9	40	6	25	5	4
VN-3541	258	I	Singleton	4	74	26	2	22	60	103	12	75	81	18
VN-3542	259	I	Singleton	Singleton	17	68	73	22	65	112	112	25	11	18
VN-3904	133	I	6	3	13	14	15	7	9	34	6	15	14	14
VN-3905	287	I	2	5	47	14	11	61	8	64	53	23	17	41
VN-3906	260	I	Singleton	Singleton	82	2	20	2	8	107	110	71	87	86
VN-3909	261	I	Singleton	Singleton	47	12	74	22	8	2	6	76	50	87
VN-3910	262	IIB	Singleton	Singleton	12	39	14	42	25	24	92	41	13	11
VN-3912	263	IIB	3	6	4	13	14	7	25	27	61	77	13	11
VN-3914	113	I	4	7	50	8	10	7	9	22	33	9	5	30
VN-3915	264	I	Singleton	Singleton	69	14	11	7	9	113	105	78	88	39
VN-3919	265	I	Singleton	1	8	41	10	3	9	114	33	15	49	44
VN-3921	266	I	Singleton	Singleton	84	38	11	23	9	34	110	5	17	49

Table S3. *Cont.*

Strain ID	MLST ST	MLST-Cluster	Clonal Complex (SLV-Level)	Clonal Complex (TLV-Level)	*glp*	*gyrB*	*mdh*	*metG*	*purM*	*dtdS*	*lysA*	*pntA*	*pyrC*	*tnaA*
VN-3922	226	IIB	Singleton	Singleton	4	13	42	42	23	67	103	13	13	11
VN-3924	268	I	Singleton	3	13	14	15	7	9	5	6	15	14	45
VN-3925	217	IIB	Singleton	Singleton	12	39	42	29	57	27	41	77	13	11
VN-3926	251	I	2	5	47	14	11	61	8	99	53	23	17	41
VN-3927	269	I	Singleton	1	8	8	10	3	9	115	17	15	49	44
VN-3928	268	I	Singleton	3	13	14	15	7	9	5	6	15	14	45
VN-3929	268	I	Singleton	3	13	14	15	7	9	5	6	15	14	45
VN-3931	270	I	Singleton	1	8	40	10	3	9	22	33	15	49	44
VN-3932	271	I	Singleton	Singleton	47	41	11	23	8	64	59	5	5	49
VN-3934	226	IIB	Singleton	Singleton	4	13	42	42	23	67	103	13	13	11
VN-3935	272	IIB	3	6	4	13	14	40	25	27	61	77	13	11
VN-3937	144	I	Singleton	Singleton	47	14	2	7	41	19	17	46	57	30
VN-3946	273	I	5	1	8	8	10	3	9	115	41	15	15	44
VN-3947	274	I	Singleton	Singleton	85	40	2	65	13	81	1	46	5	44
VN-3948	226	IIB	Singleton	Singleton	4	13	42	42	23	67	103	13	13	11
VN-3959	275	I	Singleton	Singleton	18	14	11	2	3	116	62	5	53	25
VN-3960	126	I	Singleton	1	8	8	10	3	9	22	55	15	49	44
VN-3961	133	I	6	3	13	14	15	7	9	34	6	15	14	14
VN-3962	269	I	Singleton	1	8	8	10	3	9	115	17	15	49	44
VN-3964	128	IIB	Singleton	Singleton	12	13	14	13	12	4	41	1	13	11
VN-3965	113	I	4	7	50	8	10	7	9	22	33	9	5	30
VN-3966	276	I	4	7	86	8	10	7	9	22	33	9	5	30
VN-3968	128	IIB	Singleton	Singleton	12	13	14	13	12	4	41	1	13	11
VN-3969	132	I	Singleton	8	49	40	44	7	8	22	57	15	5	50
VN-3970	277	I	Singleton	8	49	40	44	2	8	22	58	5	5	50
VN-3971	278	I	Singleton	Singleton	87	42	11	7	8	82	57	65	5	45

Table S3. *Cont.*

Strain ID	MLST ST	MLST-Cluster	Clonal Complex (SLV-Level)	Clonal Complex (TLV-Level)	*glp*	*gyrB*	*mdh*	*metG*	*purM*	*dtdS*	*lysA*	*pntA*	*pyrC*	*tnaA*
VN-3972	277	I	Singleton	Singleton	49	40	44	2	8	22	58	5	5	50
VN-3973	268	I	Singleton	3	13	14	15	7	9	5	6	15	14	45
VN-3974	279	I	Singleton	Singleton	47	69	46	7	3	107	108	79	5	88
VN-3975	280	I	5	1	8	8	10	66	9	115	41	15	15	44
VN-3976	281	IIB	Singleton	Singleton	4	13	14	29	43	24	109	32	13	11
VN-3977	281	IIB	Singleton	Singleton	4	13	14	29	43	24	109	32	13	11
VN-3978	282	I	Singleton	Singleton	52	42	2	7	8	35	4	5	4	4
VN-3979	128	IIB	Singleton	Singleton	12	13	14	13	12	4	41	1	13	11
VN-3980	269	I	Singleton	1	8	8	10	3	9	115	17	15	49	44
VN-3981	283	I	Singleton	Singleton	45	2	20	2	8	60	26	20	17	43
VN-3982	284	I	6	3	13	14	15	7	9	34	6	15	14	39
VN-5163	65	I	7	9	13	14	15	7	9	40	6	25	5	4

MLST, multilocus sequence typing; ST, sequence type; SLV, single locus variant; TLV, triple locus variant.

Table S4. Primers and probes used for PCR amplification and sequencing.

Primer Name	Specificity/Gene Target/Designation	Sequence (5′ to 3′)	Amplicon (bp)	T_a (°C)	Reference
SerE-F	specific for serovar E	TGTTGTTCTTGCCCACTCTC	665	64	[1]
SerE-R	specific for serovar E	CGCGCTTAGATTTGTCTCACC			[1]
Bt2-F	specific for biotype 2	AGAGATGGAAGAAACAGGCG	344		[1]
Bt2-R	specific for biotype 2	GGACAGATATAAGGGCAAATGG			[1]
vvhA-F	*V. vulnificus*-specific hemolysin	CGCCACCCACTTTCGGGCC	519		[1]
vvhA-R	*V. vulnificus*-specific hemolysin	CCGCGGTACAGGTTGGCGC			[1]
UtoxF	*toxR*	GASTTTGTTTGGCGYGARCAAGGTT	435	60	[2]
VvtoxR	*V. vulnificus*-specific *toxR*	AACGGAACTTAGACTCCGAC			[2]
vcg-typeC-F	virulence correlated gene clinical allele	AGCTGCCGATAGCGATCT	277	56	[3]
vcg-typeE-F	virulence correlated gene environmental allele	CTCAATTGACAATGATCT		47	[3]
vcg-typeC/E-R	virulence correlated gene	CGCTTAGGATGATCGGTG			[3]
VVA1612F	Region XII, 5′flanking region	ACCCTGATCGTTGGCTACTC	2.257	65	[4]
VVA1613R	Region XII, chondroitinase AC lyase	GGAGCGGTGTGATGGTGTTG			[4]
VVA1634F	Region XII, arylsulfatase A	TGACACCCAACCTAGACCAC	1.364	55	[4]
VVA1634R	Region XII, arylsulfatase A	ATTGATGCCAACCTGAG			[4]
VVA1612bF	Region XII, 5′flanking region	TGTGGAGAGCGGCAAGATCAAG	1.200	65	[4]
VVA1637R	Region XII, 3′flanking region	AACATCAACCAGCGAGTCGAAC			[4]
VVA1633a_F [a]	Region XII	CGTCATCACTCATGTCAAAGC	2483	60	this study
VVA1635c_R [a]	Region XII	GGTTTCATCGTCCCAAATGG			this study
VVA1633b_F [a]	Region XII	TCGAGATTGCAAACCGGACC			this study
VVA1635a_R [a]	Region XII	CGGCGTAGAGAATGATAACG			this study
VVA1635b_R [a]	Region XII	CGTACATCACATCCAACAGTTC			this study
VVA1634a_F [a]	Region XII, arylsulfatase A	GGCACGTTCGATCAACATTG			this study
VVA1634a_R [a]	Region XII, arylsulfatase A	TGATCGAACGTGCCATAGCC			this study
VVA1634b_F [a]	Region XII, arylsulfatase A	TCTATTTCGCCAACGTGAC			this study
VVA1634b_R [a]	Region XII, arylsulfatase A	GCAAAGTAATCGCGGATCTTG			this study

Table S4. *Cont.*

Primer Name	Specificity/Gene Target/Designation	Sequence (5′ to 3′)	Amplicon (bp)	T_a (ºC)	Reference
VVA1634c_F [a]	Region XII, arylsulfatase A	CCCCTATCAAACCAACAACC			this study
VVA1634d_F [a]	Region XII, arylsulfatase A	GCTGCTTTACCGATGTGCTC			this study
Vvu16S51-F [b]	16S rRNA gene	CAAGTCGAGCGGCAGCA	171	62	[5]
Vvu16S221-R [b]	16S rRNA gene	TCCTGACGCGAGAGGCC			[5]
Vvu16SA-P [b] (2091859)	16S rRNA gene type A allele	6-FAM-TGATAGCTTCGGCTCAA-MGBNFQ	probe		[5]
Vvu16SB-P [b] (2091860)	16S rRNA gene type B allele	VIC-CCCGTAGGCATCATGC-MGBNFQ	probe		[5]
nanA-F	sialic acid catabolism (SAC) cluster, aldolase,	TKATCGCCGCTCCYCATACA	745	58	[6]
nanA -R	sialic acid catabolism (SAC) cluster, aldolase,	GCAACGCCACCGTATTCAAC			[7]
Man IIA F	mannitol fermentation operon, enzyme IIA	GATGTTGGTGAACAACTTCTCTGC	243	61	[8]
Man IIA R	mannitol fermentation operon, enzyme IIA	TCTGAAGCCTGTTGGATGCC			[8]

T_a, annealing temperature. [a] used for gene sequencing; [b] used for Real-Time PCR.

Table S5. Genotypic and phenotypic characteristics of *V. vulnificus* strains from the Baltic Sea and North Sea.

Strain ID	Source	Sampling Site No. [a]	BT [b]	Serum Resistance	Mannitol Fermen-tation [c]	16S rRNA type	*vcg* Type	Region XII	*nanA*	MLST -ST	MLST Cluster	Risk Group [d]
VN-0279	B-sw	12	1	R	−	AB	E	+	+	217	IIB	2
VN-2813	N-sw	−	1	R	+	A	E	−	+	219	I	2
VN-2814	N-sw	−	1	R	+	A	E	−	+	219	I	2
VN-2961	B-sw	11	1	R	+	B	E	+	−	220	IIB	2
VN-2969	B-sw	10	1	R	+	AB	E	+	−	220	IIB	2
VN-3363	N-sd	4	1	R	+	A	E	+	−	223	I	2
VN-3364	N-sd	4	1	R	+	A	E	+	−	223	I	2
VN-3366	N-sd	4	1	R	−	A	E	−	−	224	I	1
VN-3367	N-sw	7	1	R	+	A	E	−	+	219	I	2
VN-3368	N-sw	7	1	I	+	B	C	−	+	225	IIA	2
VN-3369	N-sw	7	1	R	+	B	C	−	+	225	IIA	2
VN-3373	N-sd	5	1	R	−	A	E	−	−	227	I	1
VN-3374	N-sd	5	1	R	+	A	E	−	+	228	I	2
VN-3378	N-sw	2	1	R	−	A	E	−	+	229	I	2
VN-3379	N-sw	2	1	R	+	B	C	+	+	171	IIA	2
VN-3394	N-sd	8	1	R	+	A	E	+	+	230	I	2
VN-3398	N-sw	8	1	R	+	A	E	+	+	231	I	2
VN-3403	N-sw	8	1	R	+	A	E	+	+	230	I	2
VN-3408	N-sw	2	1	R	+	A	E	+	+	232	I	2
VN-3410	N-sw	4	1	R	−	A	E	−	+	233	I	2
VN-3411	N-sd	4	1	I	+	A	E	−	+	234	I	2
VN-3412	N-sw	7	1	R	+	A	E	−	+	235	I	2
VN-3415	N-sw	7	1	R	−	A	E	−	+	236	I	2

Table S5. *Cont.*

Strain ID	Source	Sampling Site No. [a]	BT [b]	Serum Resistance	Mannitol Fermen-tation [c]	16S rRNA Type	*vcg* Type	Region XII	*nanA*	MLST-ST	MLST Cluster	Risk Group [d]
VN-3418	N-sd	7	1	R	+	A	E	+	+	237	I	2
VN-3419	N-sw	5	1	R	+	A	E	−	+	238	I	2
VN-3426	N-sd	5	1	R	+	A	E	+	+	239	I	2
VN-3442	N-sd	1	1	R	+	A	E	+	+	239	I	2
VN-3443	N-sd	4	1	R	−	A	E	−	+	240	I	2
VN-3444	N-sd	4	1	R	+	B	C	+	−	241	IIA	2
VN-3446	N-sd	7	1	I	+	A	E	−	+	242	I	2
VN-3448	N-sw	7	1	R	+	A	E	+	+	250	I	2
VN-3451	N-sw	7	1	I	−	A	E	−	−	243	I	1
VN-3454	N-sd	7	1	S	−	A	E	−	−	244	I	1
VN-3457	N-sd	7	1	R	+	A	E	−	+	234	I	2
VN-3461	N-sw	5	1	S	−	A	E	−	−	244	I	1
VN-3465	N-sd	5	1	R	−	A	E	−	−	245	I	1
VN-3467	N-sd	5	1	R	−	A	E	−	−	246	I	1
VN-3477	N-sw	7	1	I	+	A	C	+	+	247	I	2
VN-3478	N-sd	7	1	R	+	B	C	−	+	248	IIA	2
VN-3479	N-sd	5	1	S	−	A	E	−	−	244	I	1
VN-3494	N-sw	7	1	R	+	A	E	−	−	249	I	2
VN-3496	N-sw	7	1	R	+	A	E	+	+	250	I	2
VN-3498	N-sd	4	1	R	−	A	E	−	+	240	I	2
VN-3500	N-sd	7	1	R	+	A	E	+	+	252	I	2
VN-3506	N-sw	5	1	R	−	A	E	−	+	253	I	2
VN-3518	N-sw	9	1	R	−	A	E	−	−	254	I	1

Table S5. *Cont.*

Strain ID	Source	Sampling Site No. [a]	BT [b]	Serum Resistance	Mannitol Fermen-tation [c]	16S rRNA Type	*vcg* Type	Region XII	*nanA*	MLST -ST	MLST Cluster	Risk Group [d]
VN-3529	N-sw	6	1	R	+	A	E	+	+	255	I	2
VN-3533	N-sw	8	1	R	+	A	E	−	+	255	I	2
VN-3536	N-sw	9	1	R	+	A	E	+	+	256	I	2
VN-3538	N-sw	6	1	R	+	B	E	+	+	110	IIA	2
VN-3539	N-sw	3	1	S	−	A	E	−	−	257	I	1
VN-3541	N-sw	8	1	R	+	A	E	+	+	258	I	2
VN-3542	N-sw	9	1	R	−	A	E	−	−	259	I	1
VN-3904	B-sd	20	1	R	−	A	E	−	−	133	I	1
VN-3905	B-sd	20	1	I	−	A	E	−	−	287	I	1
VN-3906	B-sd	19	1	R	−	A	E	−	−	260	I	1
VN-3909	B-sd	18	1	R	−	A	E	−	−	261	I	1
VN-3910	B-sd	18	1	R	−	AB	E	+	+	262	IIB	2
VN-3912	B-sd	17	1	R	−	AB	E	+	−	263	IIB	2
VN-3914	B-sd	16	1	I	−	A	E	−	−	113	I	1
VN-3915	B-sd	16	1	R	−	A	E	−	−	264	I	1
VN-3919	B-sd	26	1	I	−	A	E	−	−	265	I	1
VN-3921	B-sd	22	1	I	−	A	E	−	−	266	I	1
VN-3922	B-sw	21	1	R	−	AB	E	+	+	226	IIB	2
VN-3924	B-sd	21	1	R	−	A	E	−	−	268	I	1
VN-3925	B-sd	13	1	R	−	AB	E	+	+	217	IIB	2
VN-3926	B-sd	26	1	R	−	A	E	−	−	251	I	1
VN-3927	B-sd	22	1	I	−	A	E	−	−	269	I	1
VN-3928	B-sw	21	1	R	−	A	E	−	−	268	I	1

Table S5. *Cont.*

Strain ID	Source	Sampling Site No. [a]	BT [b]	Serum Resistance	Mannitol Fermen-tation [c]	16S rRNA Type	*vcg* Type	Region XII	*nanA*	MLST-ST	MLST Cluster	Risk Group [d]
VN-3929	B-sd	21	1	R	−	A	E	−	−	268	I	1
VN-3931	B-sd	21	1	R	−	A	E	−	−	270	I	1
VN-3932	B-sd	20	1	R	−	A	E	−	−	271	I	1
VN-3934	B-sd	18	1	R	−	AB	E	+	+	226	IIB	2
VN-3935	B-sd	17	1	R	−	AB	E	+	−	272	IIB	2
VN-3937	B-sd	17	1	R	−	A	E	−	−	144	I	1
VN-3946	B-sd	22	1	I	−	A	E	−	−	273	I	1
VN-3947	B-sd	22	1	R	−	A	E	−	−	274	I	1
VN-3948	B-sd	19	1	R	−	AB	E	+	+	226	IIB	2
VN-3959	B-sw	23	1	R	−	A	E	−	−	275	I	1
VN-3960	B-sw	24	1	S	−	A	E	−	−	126	I	1
VN-3961	B-sw	24	1	R	−	A	E	−	−	133	I	1
VN-3962	B-sw	23	1	S	−	A	E	−	−	269	I	1
VN-3964	B-sw	27	1	R	−	AB	E	+	+	128	IIB	2
VN-3965	B-sw	27	1	I	−	A	E	−	−	113	I	1
VN-3966	B-sw	27	1	I	−	A	E	−	−	276	I	1
VN-3968	B-sw	27	1	R	−	AB	E	+	+	128	IIB	2
VN-3969	B-sw	27	1	R	+	A	E	+	+	132	I	2
VN-3970	B-sw	23	1	R	+	A	E	+	−	277	I	2
VN-3971	B-sw	25	1	R	+	A	E	−	−	278	I	2
VN-3972	B-sw	24	1	R	+	A	E	+	−	277	I	2
VN-3973	B-sw	14	1	R	−	A	E	−	−	268	I	1
VN-3974	B-sw	15	1	I	−	A	E	−	−	279	I	1

Table S5. *Cont.*

Strain ID	Source	Sampling Site No. [a]	BT [b]	Serum Resistance	Mannitol Fermen-tation [c]	16S rRNA Type	*vcg* Type	Region XII	*nanA*	MLST -ST	MLST Cluster	Risk Group [d]
VN-3975	B-sw	15	1	R	−	A	E	−	−	280	I	1
VN-3976	B-sw	15	1	R	−	AB	E	+	+	281	IIB	2
VN-3977	B-sw	15	1	R	−	AB	E	+	+	281	IIB	2
VN-3978	B-sw	15	1	R	+	A	E	−	−	282	I	2
VN-3979	B-sd	15	1	R	−	AB	E	+	+	128	IIB	2
VN-3980	B-sw	27	1	R	−	A	E	−	−	269	I	1
VN-3981	B-sw	23	1	R	−	A	E	−	−	283	I	1
VN-3982	B-sw	27	1	R	−	A	E	−	−	284	I	1
VN-5163	B-sw	28	1	S	−	A	E	−	−	65	I	1

N, North Sea; B, Baltic Sea; sw, seawater; sd, sediment; R, resistant; I, intermediate resistant; S, susceptible; ST, sequence type. [a] Sampling site numbers shown in Figure 1. [b] Biotype assessed biochemically and by multiplex PCR. [c] Mannitol fermentation tested biochemically and by presence of mannitol fermentation operon (PCR). [d] Risk group 2 comprising strains with two or more pathogenicity markers, risk group 1 comprising strains without or with one pathogenicity marker.

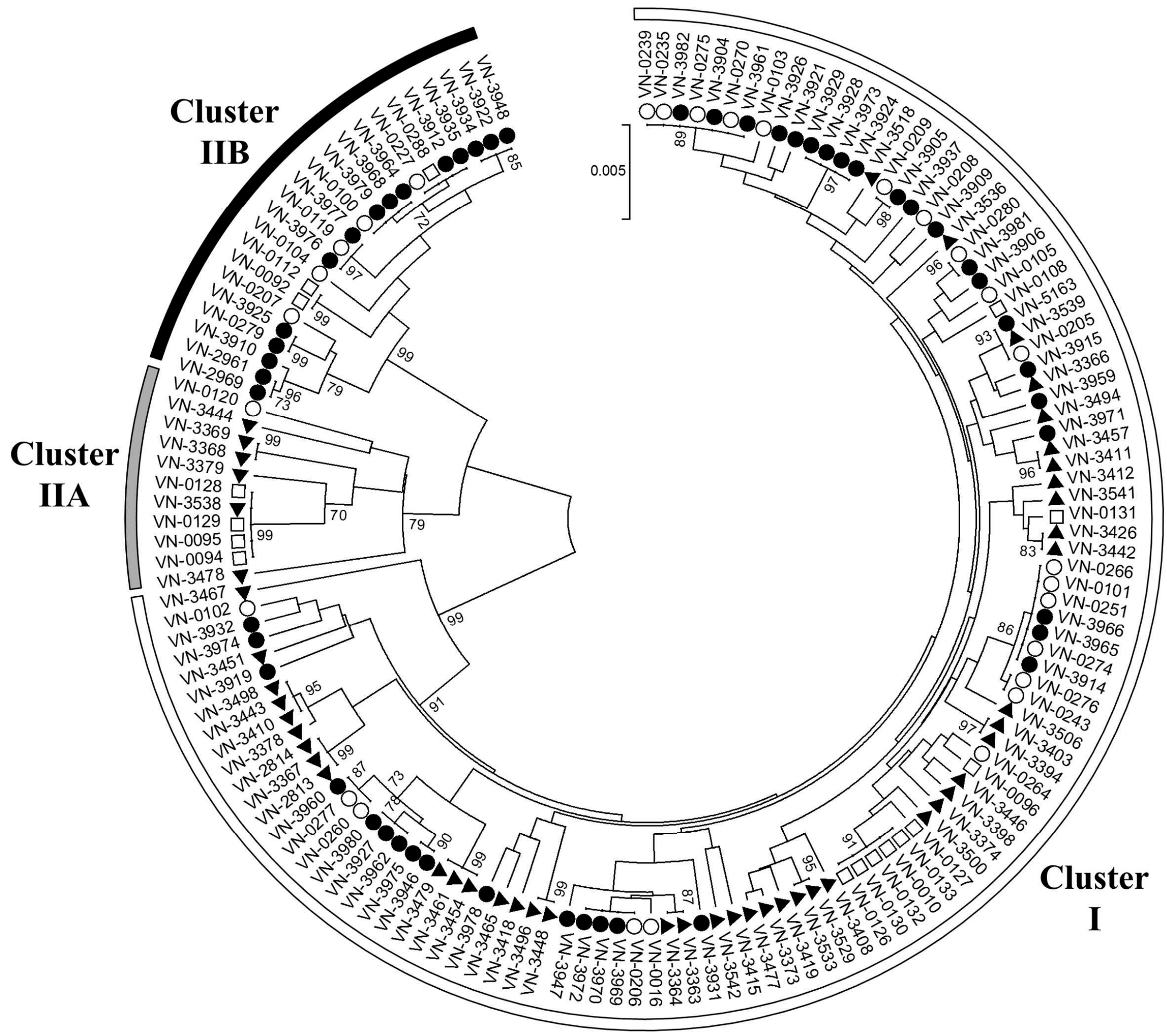

Figure S1. Population structure of *V. vulnificus* biotype 1 isolates from the North Sea (▲) and Baltic Sea (●) based on concatenated MLST sequences of three housekeeping genes (*gyrB, dtdS, and pyrC*). Bootstrap values above 70% are shown next to the branches. Semicircles around the tree highlight the association of strains to MLST cluster I (white), IIA (grey), and IIB (black). Sequences from clinical (□) and environmental (○) Baltic Sea isolates from a previous study [6] were included for comparison.

References

1 Sanjuan, E.; Amaro, C. Multiplex PCR assay for detection of vibrio vulnificus biotype 2 and simultaneous discrimination of serovar e strains. *Appl. Environ. Microbiol.* **2007**, *73*, 2029–2032.

2 Bauer, A.; Roervik, L.M. A novel multiplex pcr for the identification of *vibrio parahaemolyticus*, *vibrio cholerae* and *vibrio vulnificus*. *Lett. Appl. Microbiol.* **2007**, *45*, 371–375.

3 Rosche, T.M.; Yano, Y.; Oliver, J.D. A rapid and simple PCR analysis indicates there are two subgroups of *vibrio vulnificus* which correlate with clinical or environmental isolation. *Microbiol. Immunol.* **2005**, *49*, 381–389.

4 Cohen, A.L.V.; Oliver, J.D.; DePaola, A.; Feil, E.J.; Boyd, E.F. Emergence of a virulent clade of *vibrio vulnificus* and correlation with the presence of a 33-kilobase genomic island. *Appl. Environ. Microbiol.* **2007**, *73*, 5553–5565.
5 Vickery, M.C.L.; Nilsson, W.B.; Strom, M.S.; Nordstrom, J.L.; DePaola, A. A real-time pcr assay for the rapid determination of 16s rrna genotype in *vibrio vulnificus*. *J. Microbiol. Methods* **2007**, *68*, 376–384.
6 Bier, N.; Bechlars, S.; Diescher, S.; Klein, F.; Hauk, G.; Duty, O.; Strauch, E.; Dieckmann, R. Genotypic diversity and virulence characteristics of clinical and environmental *vibrio vulnificus* isolates from the baltic sea region. *Appl. Environ. Microbiol.* **2013**, *79*, 3570–3581.
7 Lubin, J.B.; Kingston, J.J.; Chowdhury, N.; Boyd, E.F. Sialic acid catabolism and transport gene clusters are lineage specific in *vibrio vulnificus*. *Appl. Environ. Microbiol.* **2012**, *78*, 3407–3415.
8 Froelich, B.; Oliver, J. Orientation of mannitol related genes can further differentiate strains of *vibrio vulnificus* possessing the *vcgc* allele. *Adv. Stud. Biol.* **2011**, *3*, 151–160.

© 2015 by the authors; licensee MDPI, Basel, Switzerland. This article is an open access article distributed under the terms and conditions of the Creative Commons Attribution license (http://creativecommons.org/licenses/by/4.0/).
